# A Dental Surgical Unit for the Mentally Handicapped

**Published:** 1974-01

**Authors:** J. D. Rees

**Affiliations:** Formerly Assistant Dental Surgeon Stoke Park Hospital Group, Bristol


### A Dental Surgical Unit for the Mentally Handicapped

J. D. Rees, L.D.S., R.C.S. (ENG)

Formerly Assistant Dental Surgeon
Stoke Park Hospital Group, Bristol

**Introduction**

In August 1970 a General Anaesthetic Unit, on the lines of a minor operating theatre, was established at Stoke Park Hospital, Bristol.

**Function**

Its purpose was to treat patients whose degree of subnormality was such that operative procedures with local anaesthesia and sedation would be difficult and rarely successful. Full general anaesthesia with endotracheal intubation and throat pack made it possible for the most intractable patient to have the necessary treatment completed in one visit. The theatre is equipped with all the necessary apparatus and instruments for minor oral surgery. (Whitehead, 1971).

**Theatre Staff**

A Sister-in-Charge and a Staff Nurse, both experienced in operating theatre procedures, are in attendance during sessions. An experienced consultant from a neighbouring hospital anaesthetises the patients.

**Pre-Operative Care of the Patient**

The medical staff and the consultant anaesthetist assess the fitness of patients for general anaesthesia. The anxiolytic and central muscle relaxant drug diazepam (Valium) has proved to be excellent in the pre-anaesthetic control of the restless and apprehensive subject.

Antibiotics are used selectively—mandatory in all cases of congenital and other cardiac defects.

**Post-Operative Care — Recovery Room**

A nurse who has received instructions from the consultant on the care of the unconscious patient remains on duty throughout the session. The recovery room has all the necessary apparatus and drugs for resuscitation. In all cases post-anaesthetic and post-extraction recovery has been uneventful.

**Operative Procedures**

Strict theatre asepsis is maintained. Dental treatment for this category of patient would of necessity be limited in scope:—

(1) Removal of grossly carious, unsaveable teeth and infected roots.

(2) Surgical removal of unerupted impacted third molars to avoid the painful inflammatory stages of pericoronitis. A mobile x-ray apparatus is available to aid diagnosis.

(3) Gingivectomies are performed to remove chronic hyperplastic gingival masses from patients who are receiving the drug phenytoin sodium for the control of epilepsy.

(4) Enucleation of epithelial dental cysts.

(5) Treatment of maxillary incisor teeth, the edges of which are frequently damaged when patients fall against a chair or table edge during epileptic seizure.

(6) Conservation of teeth under general anaesthesia is selective and assessed on the individual's existing standard of oral hygiene.

**CASES OF INTEREST TREATED UNDER GENERAL ANAESTHESIA**

**Fallot's Tetralogy**

A girl of 13 years had gingival recession and low-grade chronic alveolar osteitis affecting the lower central and lateral incisors. The interdental pockets were deep and suppurating. A cover of 1.2 mega units of Penidural was given 30 minutes before the four extractions, followed by a further course of procaine penicillin 600,000 units b.d. for five days. Healing was uneventful.

**Mongolism**

Invariably the lower incisors, in particular, become loose due to irreversible chronic periodontitis. Early removal of affected teeth prevented the spread of infection to posterior teeth in the mandible. 19 males and 9 females received treatment.

Abnormalities of oral cavity and dentition — predominantly microstomia, macroglossia, high narrow palate and dry fissured lips. There was a noticeable degree of skeletal Class III classification of occlusion. There were cases of partial anodontia, supernumerary teeth and retained deciduous teeth. Morphologically the anterior teeth were small and shovel-shaped and the crown of the small molars and premolars had ill-defined cusps. There were several cases of enamel hypoplasia. (Swallow, 1964).

**Mandibulo-Facial Dysostosis**

(Berry-Franceschetti Syndrome)

A male aged 64 developed advanced chronic periodontitis, necessitating the clearance of 22 teeth.

All teeth had abnormally large crowns and long slender curved roots. Both upper third molars had four roots.

The high narrow palate had ill-defined thick asymmetric pattern of the palatine rugae. Alginate impressions were taken prior to extractions to preserve permanently plaster models of the dentition of this rare case. (Jancar, 1962).

**Porphyria**

All patients requiring treatment under full general anaesthesia are screened. Sedative drugs containing barbiturates and their derivatives are strictly contra-indicated in positive reactions. One female and one male were treated. The teeth showed a red-brown discolouration.

**XXXXY Syndrome**

A male of 26 years had 4 carious teeth removed. A transalveolar resection technique had to be adopted, the x-ray films having shown abnormal root curvatures with conflicting lines of withdrawal.

The incisors were peg-shaped and the posterior teeth had poor cusp formation. (Jancar, 1964).

**Phenylketonuria**

One female aged 48 had several grossly carious teeth removed. The extractions were difficult, due to exostosed apices. The enamel was yellow with brown pigmented spots.

**Achondroplasia**

Two cases of achondroplastic dwarfs, one female aged 56 and one male aged 53, underwent total clearances.

Distinguishing features were — small maxilla, shallow palate, ill-defined palatine rugae, small crown formation, with short stunted roots.

**Prader-Willi Syndrome**

A 22 year old female had mesio-obliquely impacted lower third molars and unerupted maxillary third molars removed. Distinguishing features in this case were high narrow palate, asymetric palatine rugae and yellowish teeth. (Jancar, 1971).

**Summary**

The formation and the functioning of a Dental Surgical Unit has been described. A noteworthy result of treatment was the reports from nursing staff of improved general health and behaviour amongst patients who were relieved of dental pain and sepsis. Up to the present time 322 patients have received radical treatment for the elimination of grossly infected teeth and the correction of congenital dental defects. The chronological age ranged from 7 to 64. Average I.O. 15. Ratio of females to males 1:2.

**Acknowledgements**

I wish to thank the Hospital Management Committee, Dr. W. A. Heaton-Ward, Consultant Psychiatrist-in-Charge, Dr. J. Jancar, Consultant Psychiatrist, the Medical, Nursing and other staff of the Stoke Park Hospital Group, and Dr. I. B. Sutherland, Senior Administrative Medical Officer, South West Regional Hospital Board, for their help and encouragement in preparing this paper. Also, Dr. T. Wilton, Senior Consultant Anaesthetist, Dr. A. Diamond, Consultant Anaesthetist, the Consultant Dental Surgeons, Theatre Nursing Staff of the Facio-maxillary Unit, Sterile Supply Department—all from Frenchay Hospital, Bristol, for their invaluable aid in establishing a successful Dental Surgical Unit.

**References**

1) JANCAR, J. (1962) Mandibulo-Facial Dysostosis (Berry-Franceschetti Syndrome) associated with Severe Mental Subnormality and Consanguinity, Proc. London Conf. Scientific Study Mental Deficiency, London, **1**, 329.
2) JANCAR, J. (1964) Mentally Defective Males with XXXXY Chromosomes. Proc. Int. Copenhagen Congr. for the Scientific Study of Mental Retardation. **1**, 179.
3) JANCAR, J. (1971) Prader-Willi Syndrome (Hypotonia, Obesity, Hypogonadism, Growth and Mental Retardation). J. ment. Defic. Res. **15**, 20.
4) SWALLOW, J. N. (1964) Dental Disease in Children with Down's Syndrome, J. ment. Defic. Res. **8**, 102.
5) WHITEHEAD, F. I. H. (1971) Minor Oral Surgery in a Dental Hospital Day Stay Unit. Br. Dent. J. **130**, 69.

